# Impact of Anemia and Dual Antiplatelet Therapy on Mortality in Patients Undergoing Percutaneous Coronary Intervention with Drug-Eluting Stents

**DOI:** 10.1038/srep17213

**Published:** 2015-11-25

**Authors:** Huili Wang, Yuan Yang, Lufeng Ma, Xian Wang, Jun Zhang, Jinguo Fu, Shouyan Zhang, Ling Zhang, Dayi Hu, Rongjing Ding

**Affiliations:** 1 School of Public Health, Capital Medical University, Beijing, China; 2 School of General Practice and Continuing Education, Capital Medical University, Beijing, China; 3 Department of Cardiology, First Affiliated Hospital of Chongqing Medical University, Chongqing, China; 4 Department of Cardiology, Beijing University of Chinese Medicine Dongzhimen Hospital, Beijing, China; 5 Department of Cardiology, Cangzhou Central Hospital, Cangzhou, Hebei Province, China; 6 Department of Cardiology, Luoyang Central Hospital Affiliated to Zhengzhou University, Luoyang, Henan Province, China; 7 Department of Cardiology, Peking University People’s Hospital, Beijing, China

## Abstract

The objective was to assess the impact of baseline anemia on all-cause mortality and whether 12-month dual antiplatelet therapy (DAPT) affects 1-year mortality linked to anemia in patients after percutaneous coronary intervention (PCI) with drug-eluting stents (DES). 4109 enrolled patients divided into three groups based on their pre-procedural hemoglobin (Hb) level: Hb < 100 mg/L represented moderate-severe anemia; 100 mg/L ≤ Hb < 120 mg/L for women and 100 mg/L ≤ Hb < 130 mg/L for men represented mild anemia; Hb ≥ 120 mg/L for women and Hb≥130 mg/L for men represented no anemia. DAPT medications were prescribed when patients were discharged. There were significant differences in 30-day and 1-year mortality between moderate-severe anemia and no anemia patients (HR 8.05, 95% CI 1.46 to 44.33, *P* = 0.017; HR 3.93, 95% CI 1.11 to 13.98, *P* = 0.034), and in long-term mortality between anemia and no anemia groups (HR 1.82, 95% CI 1.17 to 2.83, *P* = 0.008 for mild anemia; HR 3.19,95% CI 1.29 to 7.86, *P* = 0.012 for moderate-severe anemia). There was not significant interaction between 12-month DAPT and anemia on mortality in anemic patients (P for interaction > 0.05). Anemia shows association with increased all-cause mortality in patients undergoing PCI. Twelve-month DAPT does not show synergy with anemia to increase the risk of all-cause 1-year mortality in anemic patients after PCI.

Anemia is common in patients with cardiovascular disease and significantly affects the mortality of patients with coronary artery disease (CAD) undergoing percutaneous coronary intervention (PCI)^1^,^2^,^3^. The use of drug-eluting stents (DES) has rapidly increased worldwide^4^ because they reduce the risk of restenosis compared with bare metal stents (BMS)^5^,^6^. The importance of dual antiplatelet therapy (DAPT) has repeatedly been stressed in the DES era due to the risk of late stent thrombosis. It is controversial whether the use of DAPT after DES increases the mortality of PCI linked with anemia. The purpose of the present analysis was to investigate the impact of pre-procedural anemia on all-cause mortality in relation to DAPT in a cohort of 4109 patients treated with DES during a follow-up period of up to 7 years.

## Methods

### Study population

We retrospectively analyzed 4109 patients with coronary artery disease who underwent initial PCI with DES at four hospitals in China (Peking University People’s Hospital, Beijing; Beijing University of Chinese Medicine Dongzhimen Hospital, Beijing; Cangzhou Central Hospital, Cangzhou, Hebei Province; Luoyang Central Hospital, Luoyang, Henan Province) from January 1st, 2006 to June 30th, 2011.

Demographics, clinical characteristics, angiographic data and hospital outcome data were derived from hospital charts. Blood samples for Hb, left ventricular ejection fraction (LVEF) and estimatedglomerular filtration rate (eGFR) were collected before PCI.

According to criteria of the World Health Organization (WHO)^7^, anemia is defined as a hemoglobin concentration of <130 mg/L for men and <120 mg/L for women. The patients were divided into three groups based on their pre-procedural hemoglobin (Hb) level: Hb < 100 mg/L represented moderate-severe anemia; 100 mg/L ≤ Hb < 120 mg/L for women and 100 mg/L ≤ Hb < 130 mg/L for men represented mild anemia; Hb ≥ 120 mg/L for women and Hb ≥ 130 mg/L for men represented no anemia.

### Procedure

All the PCI procedures were performed using femoral or radial artery access via standard techniques. All patients took 300 mg of aspirin and a 600 mg loading dose of clopidogrel before the procedure, received 8000 μ to 10000 μ of heparin intravenously during the procedure and were injected with low molecular weight heparin subcutaneously post-procedure for 5 to 7 days. Aspirin (100 mg daily) was given for a long period of time and clopidogrel (75 mg daily) was given for at least 12 months.

### Follow-up

The follow-up period was up to 84 months. Patients were actively followed-up for the ascertainment of major adverse cardiac events (MACE) (death, nonfatal repeat myocardial infarction, repeat revascularization) by trained physicians. Most patients were followed up with a phone call and the remainder went to a clinic for follow-up. The date of and reason for death of a patient were provided by their family.

All-cause mortality was used as our end point in the follow-up because some studies^8^,^9^ have shown that ascertainment bias and co-morbid illnesses might affect the attributed cause of death, so in medical outcome research, especially in retrospective studies, all-cause mortality is considered the most unbiased end point.

### Definition

Estimated glomerular filtration rate (eGFR) was calculated using the simplified Modification of Diet in Renal Disease (MDRD) equation: estimated GFR^MDRD^ (milliliter/minute) = 186 × (serum creatinine [milligrams/deciliter]^−1.154^ × age [years]) × 0.203 × (0.742 in women), and eGFR < 90 ml/min/1.72 m^2^ was defined as renal insufficiency^10^,^11^.

LVEF was determined by echocardiography and LVEF < 50% was defined as impaired cardiac function^12^,^13^.

### Statistical Analysis

The results of the quantitative variables are displayed as the mean ± SD. Categorical variables are summarized by absolute frequencies and percentages. Continuous variables at baseline were compared using one-way analysis of variance (ANOVA). The categorical variables were compared using the chi-square test. The Fisher exact test was used when more than 20% of cells with expected counts of less than 5 were observed. The Kaplan-Meier method of survival analysis was used to estimate time-related mortality, and the log-rank test was applied to compare the survival experiences of patients with different levels of Hb.

Multivariable Cox proportional hazards models for mortality across the different levels of Hb were performed. Potential confounders (with *P* < 0.05 in univariate analysis) were adjusted for this analysis.

For the statistical analysis, the statistical software SPSS 17.0 for Windows (SPSS Inc., Chicago, Illinois, USA) was used. Probability values and 95% confidence intervals (95% CI) are 2-sided. P values of 0.05 or less were considered statistically significant.

### Ethics Statement

This study was approved by the Medical Ethics Committee of Peking University People’s Hospital, Beijing, China and informed consent for follow-up was obtained from patients. Oral informed consent was given by the participants (the oral informed consent of dead patients was given by their next of kin) for their clinical records to be used in this study. All records/information of the participants were anonymized and de-identified prior to analysis. The personal details data of the participants were not publicly available. This study was conducted in accordance with procedures approved by the Medical Ethics Committee of Peking University People’s Hospital.

## Results

**Figure 1:**
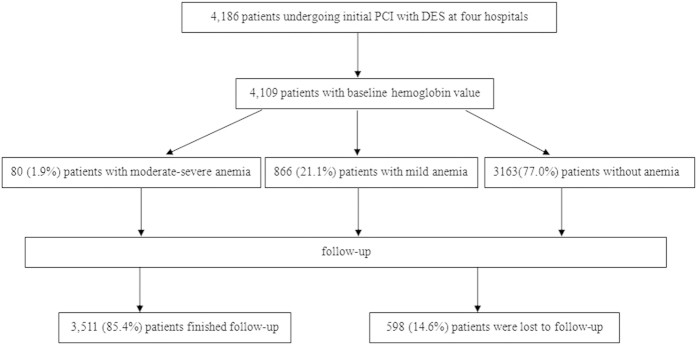
Patient flow and follow-up.

Patient flow appears in Fig. 1. Of the 4186 patients with CAD undergoing initial PCI in four hospitals, 4109 (98.2%) patients with baseline hemoglobin were included in the study, 3511 (85.4%) patients finished follow-up, and 598 (14.6%) patients were lost to follow-up because we could not make contact with them.

### Baseline Characteristics

Among the 4109 patients, 2880 (70.1%) were men and 1229 (29.9%) were women. Anemia was present in 946 (23.0%) patients. There were 866 (21.1%) mild anemia patients (100 mg/L ≤ Hb < 130 mg/L for men and 100 mg/L ≤ Hb < 120 mg/L for women) and 80 (1.9%) moderate-severe anemia patients (Hb < 100 mg/L). The mean Hb level before PCI was 136.95 ± 17.20 mg/L. The mean Hb level before PCI was 141.68 ± 15.97 mg/L in men and 125.87 ± 14.73 mg/L in women. There were 3163 (77.0%) patients considered as the non-anemic group. Baseline clinical characteristics are shown in Table 1.

Compared with non-anemic patients, anemic patients were older, more likely to be women, and more frequently had hypertension, diabetes mellitus, hyperlipidemia, and renal insufficiency. However, smoking was less prevalent in patients with anemia (*p* < 0.05 for all) (Table 1).

Angiographic features are shown in Table 2. Non-anemic and mildly anemic patients were implanted with more stents (p < 0.05 for all). There were no significant differences in antiplatelet therapy at the time of hospital discharge between anemic patients and non-anemic patients (p > 0.05 for aspirin, clopidogrel and dual antiplatelet therapy) or in the status of antiplatelet therapy medications at the end of 1 year of follow-up (p > 0.05 for aspirin, clopidogrel and dual antiplatelet therapy) (Table 3).

### Anemia and Mortality

**Figure 2:**
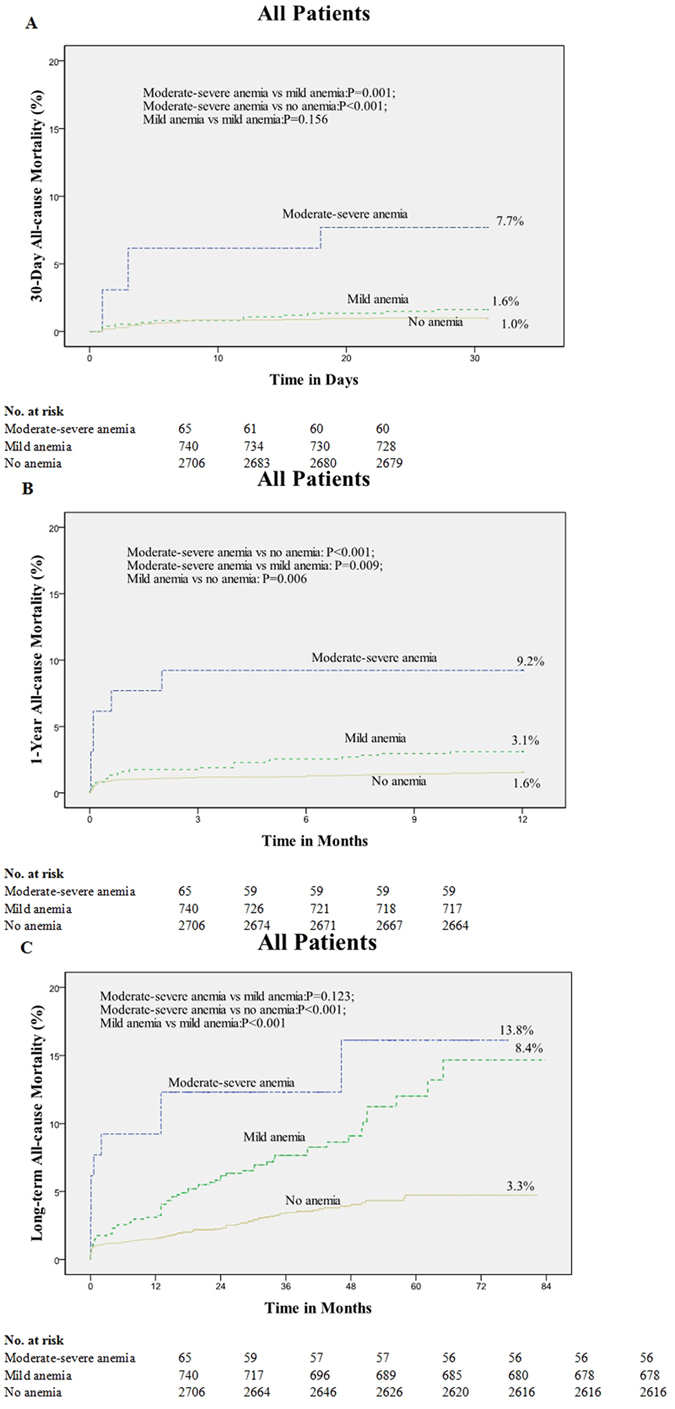
Kaplan-Meier curves for 30-day, 1-year and long-term all-cause mortality of percutaneous coronary intervention patients with different hemoglobin (Hb) levels.

Table 4 describes the all-cause mortality at 30-days, 1-year and long term in patients classified by different Hb levels. Moderate-severe anemia patients have higher 30-day and 1-year mortality compared with non-anemic patients (*P* < 0.001; *P* < 0.001) and mild anemia patients (*P* = 0.001; *P* = 0.009) (Fig. 2). The non-anemic patients and mild anemia patients only had significantly different 1-year mortality (P = 0.006) (Fig. 2).

For long-term mortality post PCI, moderate-severe anemia patients were higher than non-anemic patients (*p* < 0.001), whereas mild anemia patients had higher mortality than non-anemic patients (*p* < 0.001). The mild anemia patients and moderate-severe anemia patients did not have significantly different mortality (*p* = 0.123) (Fig. 2).

Because there were significant differences in baseline clinical characteristics, angiographic features and procedural characteristics between the anemic and non-anemic groups, the Cox proportional hazard model was performed to verify that anemia as a mortality risk factor fulfills the proportional hazard assumption. After controlling for multiple covariates including age, gender, hypertension, diabetes mellitus, hyperlipidemia, renal insufficiency, current smoking, left ventricular ejection fraction (LVEF), left circumflex artery, number of coronary artery lesions, number of stents implanted and clinical syndromes, 30-day, 1-year and long-term all-cause mortality were summarized in Table 5. Compared with no anemia, moderate-severe anemia was associated with an increased risk of all-cause 30-day (HR, 8.05; 95% CI, 1.46–44.33; P = 0.017), 1-year (HR, 3.93; 95% CI, 1.11–13.98; P = 0.034) and long-term (HR, 3.19; 95% CI, 1.29–7.86; P = 0.012) mortality in the adjusted analysis. An increased risk of all-cause death was observed for patients with mild anemia compared with patients with no anemia during long-term follow-up (HR, 1.82; 95% CI, 1.17–2.83; P = 0.008) in the adjusted analysis.

In stratified analysis across the various subgroups for 30-day, 1-year and long-term all-cause mortality, the increased risk of mortality related to anemic patients was consistent without significant interaction among patients ≥65 years of age, female patients, patients with diabetes mellitus or renal insufficiency or patients presenting with ACS (Table 6).

The baseline platelet count of 52(1.3%) patients was lower than 100 × 10^9^/L in our study. There were no significant differences in 30-day, 1-year and long-term mortality between patients with and without baseline thrombocytopenia (TC) (Table 7).

42(1.0%) patients received blood transfusion after PCI, there were no significant differences in 30-day, 1-year and long-term mortality between patients with and without blood transfusion (Table 8).

In our study, 40 (0.97%) patients underwent intra-aortic balloon pump (IABP). There were 2 (5.0%) patients with TC and 8 (20.0%) patients with post-PCI anemia (PPA) in these patients. There were no significant differences in 30-day, 1-year and long-term mortality between patients underwentIABPwith and without TC and PPA (Tables 9 and 10).

### 12-Month DAPT and Mortality

**Figure 3:**
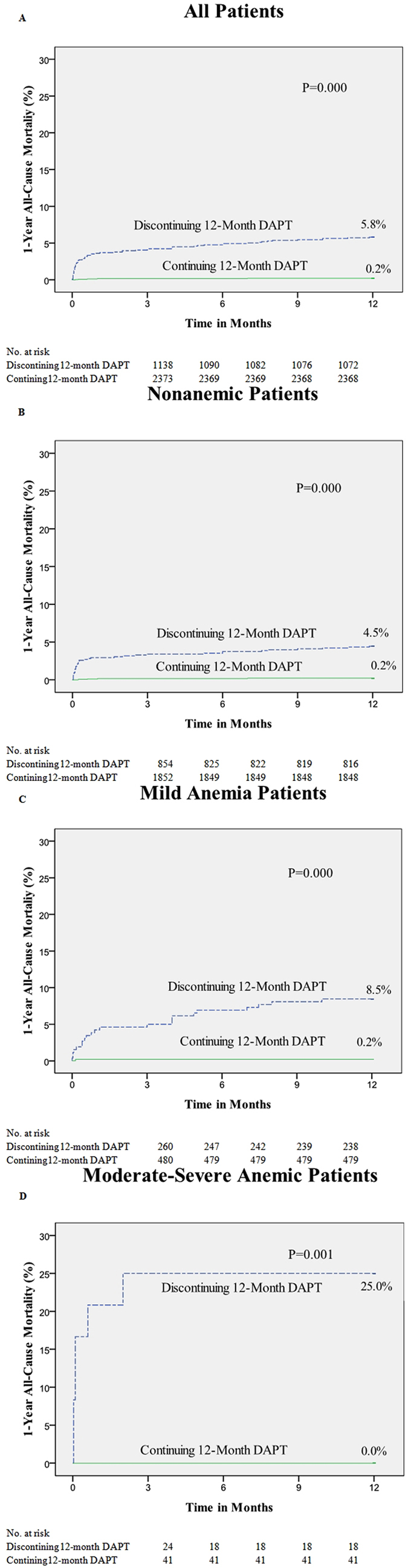
Kaplan-Meier curves for 1-year all-cause mortality of percutaneous coronary intervention patients with/without 12-month dual antiplatelet therapy by hemoglobin (Hb) group.

Discontinuing 12-month DAPT compared with continuing 12-month DAPT resulted in a significantly higher rate of all-cause mortality at 1 year in patients, including moderate-severe anemia, mild anemia and non-anemic patients (*P* = 0.002; *P* < 0.001; *P* < 0.001) (Fig. 3). In the interaction analysis between 12-month DAPT and baseline anemia, we observed no significant interaction in the relative risk for all-cause mortality in moderate-severe anemia (P for interaction = 0.986) or mild anemia (P for interaction = 0.956) patients.

## Discussion

The main findings of the present study are the following: (1) the presence of baseline anemia and the degree of anemia were strongly associated with increased mortality in patients undergoing PCI with DES; (2) compared to its absence, 12-month dual antiplatelet therapy significantly reduced all-cause mortality after PCI with DES in patients with or without anemia; and (3) there was no synergy between 12-month DAPT and anemia with respect to all-cause mortality after PCI with DES.

Many recent studies have shown associations between anemia and advanced age, diabetes mellitus, gender and chronic kidney disease^14^,^15^,^16^,^17^. Consistent with these previous reports, a similar association with advanced age, gender, diabetes mellitus and renal insufficiency in patients undergoing PCI was found in our study.

More and more studies have reported an association of anemia with 1-year and long-term mortality in patients with CAD undergoing PCI. Lee *et al*.^2^ showed that whether moderate-severe (Hb <10 mg/dL) or mild (Hb = 10 to 12 mg/dL), anemia was associated with increased 1-year mortality, and severe anemia was associated with higher risk than mild anemia. Tsujita *et al*.^18^ noted that pre-procedural anemia was strongly associated with increased 1-year mortality in patients with AMI undergoing PCI. Recently, Pilgrim *et al*.^12^ reported that severe anemia was associated with long-term mortality in a study of 6528 patients undergoing revascularization with unrestricted use of drug-eluting stents, but they did not mention the hemoglobin concentration of severe anemia in the study.

Different studies have obtained different results about the association between anemia and 30-day mortality. Some research^12^,^19^ shows no increased risk of 30-day mortality for anemic patients. Tsujita K, *et al*. and Voeltz MD, *et al*.^18^,^20^ showed that pre-procedural anemia was associated with a higher risk of 30-day mortality in patients undergoing PCI, but they did not mention the impact of the severity of anemia on 30-day mortality.

In contrast to previous studies, in our study, we only observed the association between anemia and mortality in patients with CAD undergoing PCI in men. Tsujita K *et al*.^18^ reported a similar result and explained that these divergent results might be due to differences in patients in the different studies.

The effects of degree of anemia on short- and long-term mortality were different. We only observed moderate-severe anemia to be associated with increased 30-day and 1-year mortality in patients undergoing PCI after adjustment. Not only moderate-severe anemia but also mild anemia were shown to be associated with long-term mortality.

Several proposed mechanisms can be held responsible for the observed increase in mortality among anemic patients with CAD undergoing PCI. Anemia impairs myocardial oxygen delivery and results in myocardial ischemia. Then, cardiac output increases as an adaptive physiological mechanism. The increased cardiac output leads to the development of left ventricular hypertrophy. Anemia causes an increase in mass of the left ventricle and volume dilation of the left ventricular end-diastolic phase. These changes result in an increase of the risk of arrhythmia, MI, and myocardial fibrosis^21^,^22^. Some studies^23^,^24^ have reported that in the anemic state, the recruitment of endothelial cells and cardiac stem cells, which may come from bone marrow and be mobilized during cardiac injury, may be impaired after cardiac injury.

Alternatively, the higher frequency of comorbidities in anemic patients suggests that anemia might be a marker of disease severity. In other words, anemia might be the effect of some underlying chronic disease that could affect mortality among patients with CAD undergoing PCI.

The use of DES increases the risk of late stent thrombosis^25^,^26^, and stopping dual antiplatelet therapy prematurely after DES is a strong independent predictor of stent thrombosis and late events^27^,^28^. The optimal duration of DAPT after PCI with DES is still controversial. The European Society of Cardiology (ESC) guidelines recommend only up to 6–12 months of DAPT after PCI with DES^29^,^30^, but the American Heart Association/American College of Cardiology guidelines recommends 12 months of DAPT after DES^31^. Chinese clinical practice guidelines also recommend at least 12 months of DAPT after DES^32^.

Many studies have reported that anemia increases the risk of hemorrhagic complications after PCI^12^,^33^,^34^. Some researches showed that prolonged use of DAPT for 1 year increases the rate of major bleeding events after PCI^35^,^36^, both baseline anemia and bleeding increase mortality^33^. Therefore, the use of 12-month DAPT in anemic patients, especially in severely anemic patients, has been considered to further increase mortality after PCI linked anemia. Therefore, Pilgrim T^12^ suggested that BMS should be used instead of DES in severely anemic patients to avoid the prolonged use of DAPT.

However, a study of 1816 patients^37^ reported that 1-year DAPT after PCI did not increase the overall rate of major or minor bleeding. A systematic review and meta-analysis by Elmariah S *et al*.^38^ also showed that extended duration DAPT was not associated with the risk of all-cause mortality compared with short DAPT.

These previous studies did not evaluate the all-cause mortality in anemic patients with or without 12-month DAPT and whether anemia or 12-month DAPT had synergistic effects on all-cause mortality after PCI with DES.

In the present study, the total all-cause mortality of patients continuing 12-month DAPT was significantly lower than that of patients discontinuing 12-month DAPT. To know whether similar results are obtained in anemic patients, stratified analysis according to the degree of anemia was performed. In both the moderate-severe and mild anemia groups, the mortality of patients with 12-month DAPT was remarkably lower than that of patients without 12-month DAPT. Then, we performed interaction analysis to understand whether the use of 12-month DAPT synergized with anemia on 1-year all-cause death, and the results showed there were no significant differences between the three groups. This means that the use of 12-month DAPT in anemic patients, even in severely anemic patients, did not increase the impact of anemia on all-cause 1-year mortality after PCI with DES; there was no synergy between 12-month DAPT and anemia. Therefore, we consider, for those anemic patients without a history of active bleeding before PCI, DES and 12-month DAPT might be chosen by physicians.

In clinical practice, blood transfusions sometimes will be used to increase hemoglobin level of anemic patients. A recent meta-analysis study^39^ reported blood transfusions increased risk of mortality after PCI. In our study, 42(1.0%) patients received blood transfusion. We did not find blood transfusions increased risk of mortality after PCI in our study. This may be due to the small number of patients with transfusion in our study.

Sattur S *et al*.^40^ found post-PCI anemia (PPA) was common in PCI patients and PPA increased long-term mortality. Thus, they thought PPA might be a stronger marker of adverse outcomes than anemia prior to PCI. But in the study of Sattur S *et al*., the proportion of patients with anemia prior to PCI in patients with PPA was not mentioned, and there was significant difference in baseline Hb between patients with PPA and without PPA (12.1 ± 1.3 gm/dL vs. 14.1 ± 1.4 gm/dL, *P* < 0.0001). To the contrary, our study aimed to evaluate the impact of baseline anemia and DAPT with the short and long-term mortality, unfortunately we didn’t evaluate PPA. PPA might be an independent risk factor for long-term mortality after adjusting baseline characteristics (including baseline Hb, adjusted OR: 1.3, 95% CI: 1.1–1.6, *P* = 0.023), but whether PPA or baseline anemia plus PPA is a stronger predictor than baseline anemia should be discussed in future researches.

Previous researches^41^,^42^ showed thrombocytopenia (TC) is associated with increased risk of bleeding and ischemic events. Studies also showed that intra-aortic balloon pump (IABP) was a cause of both TC^43^ and PPA^40^ and influenced the long-term ischemic events. Although the significant difference in mortality between patients with and without baseline TC was not found in our study, the change of platelet count following IABP should be taken into account in the process of DAPT. There were only 2 patients with TC and 8 patients with PPA in 40 patients underwent IABP in our study. We found mortality was associated with neither TC nor PPA in patients with IABP. This may be caused by the small amount of patients underwent IABP and the low incidence of TC and PPA.

### Limitations

There are some limitations in our study. This study is a retrospective cohort study and thus has the characteristics of a retrospective analysis; therefore, the results of the study require confirmation in a prospective study in the future. The cause of anemia in the patients in our study was not known, and the observed association between anemia and mortality in this observational study may be influenced by different etiologies. In present study, patients did not have conventional post-PCI blood test because they were discharged at 1–2 days after PCI, unless obvious bleeding and complication was found. So we did not collect the data of Hb and platelet count post-PCI from history record and assess the relationship between PPA/TC and mortality after PCI. Further studies will be clearly needed to investigate these problems.

## Conclusions

Anemia is a common problem in patients undergoing PCI, and it showed associated with increased all-cause mortality patients undergoing PCI with DES. Therefore, hemoglobin levels should be routinely considered as a risk that increases mortality in patients undergoing PCI with DES. Twelve-month DAPT did not show synergy with anemia to increase the risk of all-cause 1-year mortality in anemic patients after PCI with DES, especially moderate-severe anemia patients. Anemic patients, especially those with moderate-severe anemia, might be considered to undergo PCI with DES and receive 12-month DAPT. Due to the nature of retrospective cohort study and analysis, the results of this study require confirmation in further prospective random control test.

## Additional Information

**How to cite this article**: Wang, H. *et al*. Impact of Anemia and Dual Antiplatelet Therapy on Mortality in Patients undergoing Percutaneous Coronary Intervention with Drug-Eluting Stents. *Sci. Rep*. **5**, 17213; doi: 10.1038/srep17213 (2015).

## Figures and Tables

**Table 1:** Baseline Characteristics.

Baseline Characteristics	No Anemia	Mild Anemia	Moderate-severe Anemia	*P* Value
Total, n	3163(77.0)	866(21.1)	80(1.9)	
Clinical features				
Age, y, (SD)	63.34 ± 8.81	66.93 ± 10.07	69.87 ± 11.24	<0.001
Male gender, n (%)	2305(72.9)	547(63.2)	28(35.0)	<0.001
Hypertension, n (%)	1893(59.8)	536(61.9)	58(72.5)	0.049
Diabetes mellitus, n (%)	722(22.8)	259(29.9)	25(31.3)	<0.001
Current smoker, n (%)	1591(50.3)	333(38.5)	16(20.0)	<0.001
Hyperlipidemia, n (%)	484(15.3)	100(11.5)	10(12.5)	0.016
Previous heart failure, n (%)	203(6.4)	61(7.0)	4(5.0)	0.669
Hb, mg/L,	143.41 ± 12.74	117.86 ± 7.83	88.28 ± 12.28	<0.001
eGFR, <90 ml/min/1.72 m^2^, n (%)	72(2.3)	62(7.2)	20(25.0)	<0.001
LVEF, <50%, n (%)	196(6.2)	81(9.4)	12(15.0)	<0.001
Clinical syndromes				<0.001
ACS	2274(71.9)	767(88.6)	67(83.8)	
Non-ACS	889(28.1)	99(11.4)	13(16.2)	

SD indicates standard deviation; eGFR, estimated glomerular filtration rate; LVEF, left ventricular ejection fraction; ACS, acute coronary syndrome.

**Table 2:** Procedural Characteristics.

Procedural Characteristics	No Anemia	Mild Anemia	Moderate-severe Anemia	*P* Value
Infarct related artery				
Left main artery, n (%)	217 (6.9)	67 (7.7)	6 (7.5)	0.664
Left anterior descending artery, n (%)	2753 (87.0)	763 (88.1)	70 (87.5)	0.704
Left circumflex artery, n (%)	1915 (60.5)	567 (65.5)	42 (52.5)	0.008
Right coronary artery, n (%)	2004 (63.4)	581 (67.1)	49 (61.3)	0.111
No. of coronary artery lesions, n(SD)	2.1 (0.8)	2.2 (0.8)	2.0 (0.9)	0.003
No. of more than 20 mm coronary artery lesion length, n(SD)	1.1 (1.3)	1.1 (1.3)	1.2 (1.3)	0.700
No. of stents implanted, n(SD)	1.9 (1.2)	1.9 (1.2)	1.6 (1.1)	0.039

**Table 3:** Dual Antiplatelet Therapy (DAPT) Medications at Discharge and the End of 1-year Follow-up.

	No Anemia	Mild Anemia	Moderate-severe Anemia	*P* Value
Medications at discharge				
Aspirin, n (%)	3163 (100.0)	866 (100.0)	80 (100.0)	—
Clopidogrel, n (%)	3156 (99.8)	864 (99.8)	80 (100.0)	1.000
Dual antiplatelet therapy, n (%)	3156 (99.8)	864 (99.8)	80 (100.0)	1.000
Medications at end of 1-year follow-up				
Aspirin, n (%)	2392 (88.4)	611 (82.6)	48 (73.8)	<0.001
Clopidogrel, n (%)	2115 (78.2)	583 (78.8)	56 (86.2)	0.292
Dual antiplatelet therapy, n (%)	1852 (68.4)	480 (64.9)	41 (63.1)	0.135

^*^The number of patients at end of follow-up was 3511.

**Table 4:** Thirty-day, 1-year and Long-term Clinical Outcomes after PCI.

	No Anemia	Mild Anemia	Moderate-severe Anemia	*P* Value
30-Day death, n (%)	27 (1.0)	12 (1.6)	5 (7.7)	<0.001
1-Year outcomes				
All-cause death, n (%)	42 (1.6)	23 (3.1)	6 (9.2)	<0.001
Reinfarction, n (%)	59 (2.2)	19 (2.6)	2 (3.1)	0.600
Repeated revascularization, n (%)	97 (3.6)	27 (3.6)	3 (4.6)	0.797
Long-term outcomes				
All-cause death, n (%)	90 (3.3)	62 (8.4)	9 (13.8)	<0.001
Reinfarction, n (%)	104 (3.8)	30 (4.0)	3 (4.6)	0.846
Repeated revascularization, n (%)	117 (4.3)	34 (4.6)	4 (6.2)	0.655

^*^The number of patients at end of follow-up was 3511.

**Table 5:** Adjusted 30-day, 1-year and Long-term Mortality Hazard Ratio for Anemia.

		Hazard Ratio for Mortality		
		HR	95% CI	*P* value
30-Day mortality				0.054
	mild anemia*	1.83	0.62–5.40	0.272
	moderate-severe anemia*	8.05	1.46–44.33	0.017
1-Year mortality				0.081
	mild anemia	1.54	0.79–3.03	0.209
	moderate-severe anemia	3.93	1.11–13.98	0.034
Long-term mortality				0.005
	mild anemia	1.82	1.17–2.83	0.008
	moderate-severe anemia	3.19	1.29–7.86	0.012

CI = confidence interval.

^*^Compared with the no anemia group; age, gender, hypertension, diabetes mellitus, hyperlipidemia, renal insufficiency, current smoking, left ventricular ejection fraction (LVEF), left circumflex artery, number of coronary artery lesions, number of stents implanted and clinical syndromes were adjusted.

**Table 6:** Stratified Analysis of 30-day, 1-year and Long-term All-cause Mortality across Several Subgroups.

	Moderate-severe Anemia	Mild Anemia	No Anemia	*P*Value	*P* Interaction
30-Day overall mortality					
Age, n (%)					0.804
<65 years	0 (0)	5 (1.9)	7 (0.4)	0.021	
≥65 years	5 (10.0)	7 (1.5)	20 (2.0)	<0.001	
Gender, n (%)					0.705
Men	3 (15.0)	7 (1.5)	16 (0.8)	<0.001	
Women	2 (4.4)	5 (1.8)	11 (1.5)	0.308	
Diabetes mellitus, n (%)					0.930
No	4 (8.9)	9 (1.8)	20 (1.0)	<0.001	
Yes	1 (5.0)	3 (1.3)	7 (1.1)	0.296	
Renal insufficiency, n (%)					0.956
No	0 (0)	7 (1.5)	10 (0.5)	0.049	
Yes	5(16.1)	5 (2.0)	14 (2.3)	<0.001	
Smoking, n (%)					0.586
No	5 (9.4)	8 (1.8)	13 (1.0)	<0.001	
Yes	0 (0.0)	4(1.4)	13 (1.0)	0.750	
Acute coronary syndrome,n (%)					0.983
No	1 (12.5)	2 (4.0)	0 (0)	0.001	
Yes	3 (5.7)	10 (1.5)	25 (1.0)	0.007	
1-Year overall mortality					
Age, n (%)					0.968
<65 years	0 (0)	7 (2.6)	11 (0.7)	0.009	
≥65 years	6 (12.0)	16 (3.4)	31 (2.9)	0.001	
Gender, n (%)					0.828
Men	4 (20.0)	15 (3.2)	28 (1.4)	<0.001	
Women	2 (4.4)	8 (2.9)	14 (1.9)	0.368	
Diabetes mellitus, n (%)					0.481
No	4 (8.9)	18 (3.6)	28 (1.3)	<0.001	
Yes	2 (10.0)	5 (2.2)	14 (2.3)	0.071	
Renal insufficiency, n (%)					0.580
No	1 (3.0)	14 (3.0)	20 (1.0)	0.003	
Yes	5 (16.1)	8 (3.2)	18 (3.0)	0.000	
Smoking, n (%)					0.344
No	5 (9.4)	15 (3.3)	18 (1.3)	<0.001	
Yes	1 (8.3)	8 (2.8)	23 (1.7)	0.127	
Acute coronary syndrome,n (%)					0.366
No	2 (25.0)	3 (6.0)	1 (0.5)	<0.001	
Yes	3 (5.7)	20 (3.0)	38 (1.6)	0.008	
Long-term overall mortality					
Age, n (%)					0.965
<65 years	0 (0.0)	11 (4.1)	23 (1.4)	0.008	
≥65 years	9 (18.0)	51 (10.8)	67 (6.3)	<0.001	
Gender, n (%)					0.160
Men	5 (25.0)	44 (9.5)	65 (3.3)	<0.001	
Women	4 (8.9)	18 (6.5)	25 (3.4)	0.036	
Diabetes mellitus, n (%)					0.725
No	5 (11.1)	40 (7.9)	58 (2.8)	<0.001	
Yes	4 (20.0)	22 (9.6)	32 (5.2)	0.003	
Renal insufficiency, n (%)					0.392
No	1 (3.0)	29 (6.2)	47 (2.3)	<0.001	
Yes	8 (25.8)	32 (12.7)	37 (6.2)	<0.001	
Smoking, n (%)					0.055
No	6 (11.3)	35 (7.7)	34 (2.5)	<0.001	
Yes	3 (25.0)	27 (9.6)	55(4.1)	<0.001	
Acute coronary syndrome,n (%)					0.225
No	2 (25.0)	7 (14.0)	2 (1.0)	<0.001	
Yes	6 (11.3)	54 (8.2)	81 (3.4)	<0.001	

^*^Probability values for interaction between the effects of anemia and patients characteristics are shown for age (≥65 years), female patients, diabetes mellitus, renal insufficiency, smoking and acute coronary syndrome (ACS).

**Table 7:** Thirty-day, 1-year and Long-term Mortality in Patients With or Without Baseline Thrombocytopenia.

	Thrombocytopenia	Without Thrombocytopenia	*P* Value
30-Day mortality, n (%)	1 (2.3)	41 (1.2)	0.416
1-Year mortality, n (%)	2 (4.5)	67 (2.0)	0.217
Long-term mortality, n (%)	2 (4.5)	155 (4.5)	1.000

*The number of patients at end of follow-up was 3511. There were 42 patients without baseline platelet count.

**Table 8:** Thirty-day, 1-year and Long-term Mortality in Patients With or Without BloodTransfusion after PCI.

	Blood transfusion	Without blood transfusion	*P* Value
30-Day mortality, n (%)	2 (5.4)	42 (1.2)	0.078
1-Year mortality, n (%)	2 (5.4)	69 (2.0)	0.171
Long-term mortality, n (%)	4 (10.8)	157 (4.5)	0.087

^*^The number of patients at end of follow-up was 3511.

**Table 9:** Thirty-day, 1-year and Long-term Mortality in Patients Underwent IABP With or Without Thrombocytopenia after PCI.

	Thrombocytopenia	Without Thrombocytopenia	*P* Value
30-Day mortality, n (%)	1 (50.0)	7 (18.9)	0.372
1-Year mortality, n (%)	1 (50.0)	9 (24.3)	0.452
Long-term mortality, n (%)	1 (50.0)	14 (37.8)	1.000

^*^The number of patients underwent IABP was 40. The number of patients underwent at end of follow-up was 39. IABP indicates intra-aortic balloon pump.

**Table 10:** Thirty-day, 1-year and Long-term Mortality in Patients Underwent IABP With or Without Post-PCI Anemia.

	Post-PCI Anemia	Without Post-PCI Anemia	*P* Value
30-Day mortality, n (%)	3 (37.5)	5 (16.1)	0.323
1-Year mortality, n (%)	3 (37.5)	7 (22.6)	0.399
Long-term mortality, n (%)	5 (62.5)	10 (32.3)	0.124

^*^The number of patients underwent IABP was 40. The number of patients underwent at end of follow-up was 39. IABP indicates intra-aortic balloon pump.
